# A bioinformatic approach to identify confirmed and probable CRISPR–Cas systems in the *Acinetobacter calcoaceticus*–*Acinetobacter baumannii* complex genomes

**DOI:** 10.3389/fmicb.2024.1335997

**Published:** 2024-04-09

**Authors:** Jetsi Mancilla-Rojano, Víctor Flores, Miguel A. Cevallos, Sara A. Ochoa, Julio Parra-Flores, José Arellano-Galindo, Juan Xicohtencatl-Cortes, Ariadnna Cruz-Córdova

**Affiliations:** ^1^Posgrado en Ciencias Biológicas, Facultad de Medicina, Universidad Nacional Autónoma de México, Mexico, Mexico; ^2^Laboratorio de Investigación en Bacteriología Intestinal, Unidad de Enfermedades Infecciosas, Hospital Infantil de México Federico Gómez, Secretaría de Salud, Mexico, Mexico; ^3^Department of Biochemistry, University of Cambridge, Cambridge, United Kingdom; ^4^Centro de Ciencias Genómicas, Programa de Genómica Evolutiva, Universidad Nacional Autónoma de México, Cuernavaca, Mexico; ^5^Department of Nutrition and Public Health, Universidad del Bío-Bío, Chillán, Chile; ^6^Unidad de Investigación en Enfermedades Infecciosas, Hospital Infantil de México Federico Gomez, Mexico, Mexico

**Keywords:** *Acinetobacter baumannii*, *Acinetobacter calcoaceticus–Acinetobacter baumannii* complex, *cas* genes, CRISPR systems, prophages

## Abstract

**Introduction:**

The *Acinetobacter calcoaceticus–Acinetobacter baumannii* complex, or Acb complex, consists of six species: *Acinetobacter baumannii, Acinetobacter calcoaceticus, Acinetobacter nosocomialis, Acinetobacter pittii, Acinetobacter seifertii*, and *Acinetobacter lactucae*. *A. baumannii* is the most clinically significant of these species and is frequently related to healthcare-associated infections (HCAIs). Clustered regularly interspaced short palindromic repeat (CRISPR) arrays and associated genes (*cas*) constitute bacterial adaptive immune systems and function as variable genetic elements. This study aimed to conduct a genomic analysis of Acb complex genomes available in databases to describe and characterize CRISPR systems and *cas* genes.

**Methods:**

Acb complex genomes available in the NCBI and BV-BRC databases, the identification and characterization of CRISPR-Cas systems were performed using CRISPRCasFinder, CRISPRminer, and CRISPRDetect. Sequence types (STs) were determined using the Oxford scheme and ribosomal multilocus sequence typing (rMLST). Prophages were identified using PHASTER and Prophage Hunter.

**Results:**

A total of 293 genomes representing six Acb species exhibited CRISPR-related sequences. These genomes originate from various sources, including clinical specimens, animals, medical devices, and environmental samples. Sequence typing identified 145 ribosomal multilocus sequence types (rSTs). CRISPR–Cas systems were confirmed in 26.3% of the genomes, classified as subtypes I-Fa, I-Fb and I-Fv. Probable CRISPR arrays and *cas* genes associated with CRISPR–Cas subtypes III-A, I-B, and III-B were also detected. Some of the CRISPR–Cas systems are associated with genomic regions related to Cap4 proteins, and toxin–antitoxin systems. Moreover, prophage sequences were prevalent in 68.9% of the genomes. Analysis revealed a connection between these prophages and CRISPR–Cas systems, indicating an ongoing arms race between the bacteria and their bacteriophages. Furthermore, proteins associated with anti-CRISPR systems, such as AcrF11 and AcrF7, were identified in the *A. baumannii* and *A. pittii* genomes.

**Discussion:**

This study elucidates CRISPR–Cas systems and defense mechanisms within the Acb complex, highlighting their diverse distribution and interactions with prophages and other genetic elements. This study also provides valuable insights into the evolution and adaptation of these microorganisms in various environments and clinical settings.

## 1 Introduction

There are more than 50 Acinetobacter species. The *Acinetobacter calcoaceticus–Acinetobacter baumannii* complex or Acb complex is formed by six species that are phenotypically indistinguishable*: Acinetobacter baumannii, Acinetobacter calcoaceticus, Acinetobacter nosocomialis, Acinetobacter pittii, Acinetobacter seifertii*, and *Acinetobacter lactucae* (Villalon et al., 2015).

The Acb complex includes opportunistic pathogens, mainly related to healthcare-associated infections (HCAIs), multidrug-resistant phenotypes, and resistance to desiccation and disinfectants (Manchanda et al., 2010). Other authors have described epidemiological differences among these species, although they frequently share hospital environments (Wisplinghoff et al., 2012; Calix et al., 2019). *A. baumannii* is the main species with the most clinical importance; it is commonly isolated in intensive care units and is related to illness types such as ventilator-associated pneumonia, meningitis, bloodstream, and urinary tract infections (Moradi et al., 2015). *A. baumannii* is resistant to a broad spectrum of antibiotics, limiting therapeutic options. Recently, *A. baumannii* has become resistant to carbapenems and has been included in the list of priority pathogens resistant to antibiotics published by the World Health Organization (World Health Organization, 2017).

The *A. baumannii* genome shows great plasticity, which exposes it not only to rapid changes due to mutations but also to the transfer and acquisition of exogenous material (Yakkala et al., 2019), that is, the dynamic conversion of genetic information for acquisition and removal (Bondy-Denomy and Davidson, 2014). *A. baumannii* acquires genetic material through bacteriophages via a transduction mechanism (Chevallereau et al., 2022).

Bacteria can develop adaptive defense mechanisms against when exposed to different exogenous genetic material. The clustered regularly interspaced short palindromic repeats (CRISPR)–CRISPR-associated enzyme (Cas) system has also been associated with the acquisition of mobile genetic material and is an adaptive mechanism of immunity and resistance used by bacteria and archaea against bacteriophages and/or plasmids (Dy et al., 2013; Makarova et al., 2020).

CRISPR systems comprise palindromic elements interspersed with sequences of constant length (Mojica and Montoliu, 2016) flanking the spacers acquired from bacteriophages, plasmids, or any genetic material. The CRISPR–Cas system comprises two main components: a guide RNA (crRNA or gRNA) and genes that encode Cas proteins, which are essential for the adaptation and incorporation of genetic material (Koonin et al., 2017).

The mechanism of action of the CRISPR–Cas system involves three stages: acquisition, expression, and interference. In the acquisition stage, elements that carry genetic material are identified and recognized (protospacer); this information is cut and integrated into the CRISPR locus at the 5′ end followed by the leader sequence. In the expression stage, the sequences that are added to the CRISPR locus are recognized as spacers, which are expressed in the form of primary transcripts or precrRNAs and are cut into smaller fragments (crRNAs) through endonucleases (Waddington et al., 2016). Finally, in the interference stage, when the bacterium receives exogenous genetic material, the crRNA accompanied by Cas proteins binds via base complementarity to the previously acquired sequence, signaling to the nucleases that the external genetic element must cleave (Bhaya et al., 2011). Moreover, the CRISPR system mediates the transfer of genetic material between genomes (Marraffini and Sontheimer, 2008; O'Meara and Nunney, 2019). Genomes displaying a CRISPR array but lacking associated cas genes, it is proposed that bacteria could capture information from bacteriophages and incorporate it into their genome as a prophage. In contrast, bacteria without a CRISPR-Cas system can be infected by bacteriophages, acquiring information that encodes genes linked to resistance and virulence, thereby enhancing their adaptive capacity (Leungtongkam et al., 2020).

CRISPR–Cas IFb has been proposed as a method for subtyping *A. baumannii* strains, allowing identification of the route of origin and dissemination (Touchon et al., 2014; Karah et al., 2015). The CRISPR–Cas system identified in *A. baumannii* is characterized by the presence of the *cas1, cas2, cas3, cas5, cas7*, and *cas8* genes, which have been identified in chromosome and plasmid sequences (Mangas et al., 2019). A few studies have provided information about the prevalence of these systems in clinical strains of the Acb complex. This study aimed to perform genomic analysis of the Acb complex to identify and characterize CRISPR arrays and/or the *cas* genes, using bioinformatics programs.

## 2 Materials and methods

**Figure 1:**
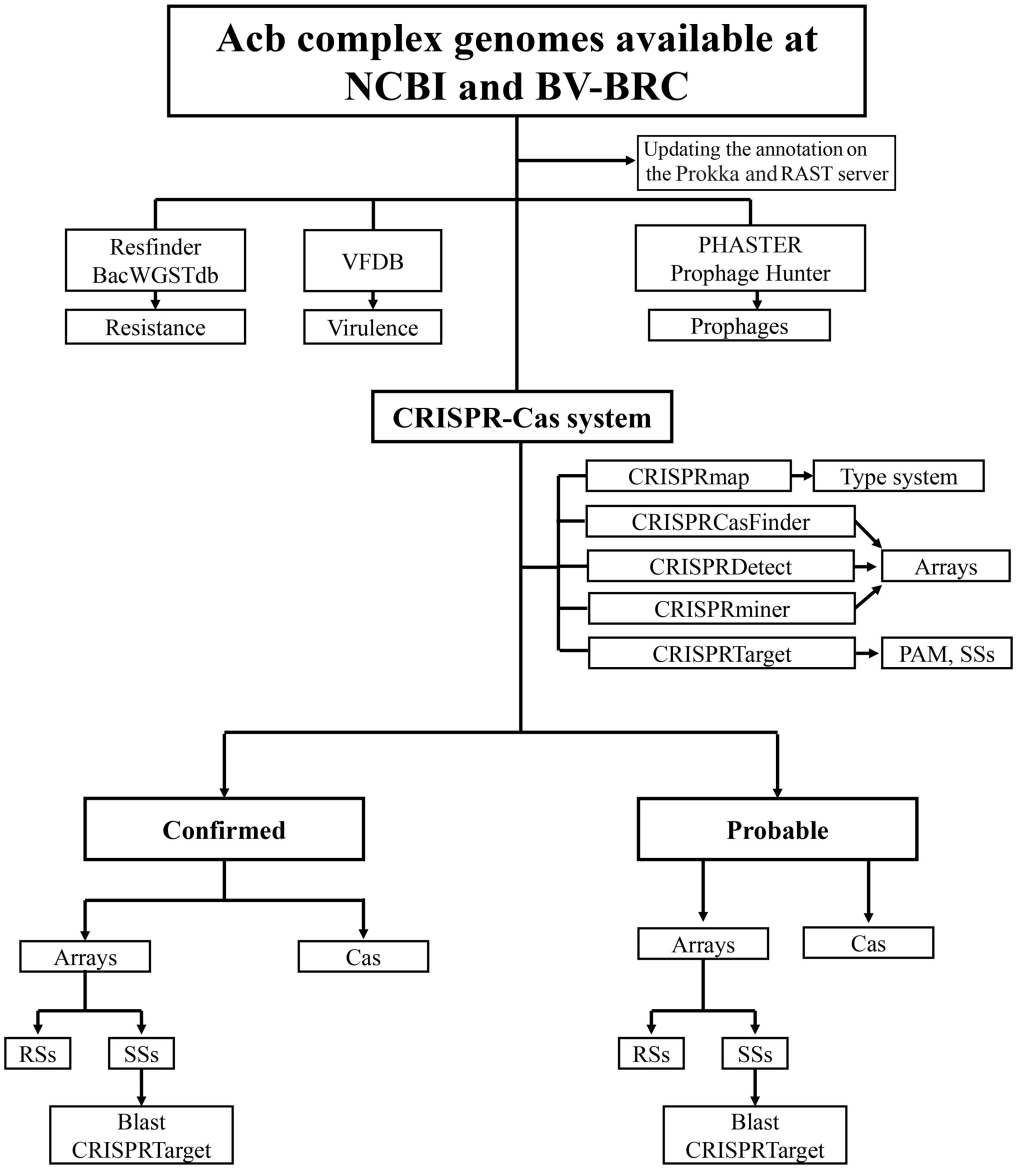
Flowchart for the search for CRISPR–Cas systems. Flowchart indicating data collection for Acb complex genomes, detection and analysis of the CRISPR–Cas systems, resistance and virulence genes, and prophage-associated sequences.

A flowchart for data collection for the Acb complex genomes, detection and analysis of the CRISPR–Cas systems is shown in Figure 1.

### 2.1 Genome database

A total of 7,743 genomes, described as “complete” (1,144) and “draft” (6,599), including 952 plasmids of the Acb complex from the National Center for Biotechnology Information (NCBI), and Bacterial and Viral Bioinformatics Resource Center (BV-BRC) databases (https://www.ncbi.nlm.nih.gov/and https://www.bv-brc.org/; accessed on September 2022), were included to screen for sequences associated with CRISPR–Cas systems. These were from the following species: *A. baumannii* (6,824), *A. nosocomialis* (246), *A. pittii* (435), *A. calcoaceticus* (31), *A. seifertii* (200), and *A. lactucae* (7).

The metadata of each strain were manually extracted, including the date, GenBank accession numbers, species, genetic material, strain ID, year of isolation, associated disease, sample, host, collection place, rST, ST, origin, site of isolation, and size (Supplementary Table 1).

#### 2.1.1 MLTS and rMLST analysis

Allele designation for sequence typing (ST) in chromosomal genomes was performed by ribosomal multilocus sequence typing (rMLST) (https://pubmlst.org/bigsdb?db=pubmlst_rmlst_seqdef_kiosk) (Jolley et al., 2018). The ST and Clonal Complexes (CC) have been determined using the Oxford scheme (Bartual et al., 2005).

### 2.2 Bioinformatics search of CRISPR-Cas systems

The search for CRISPR systems was carried out in the chromosome and plasmid sequences using the programs CRISPRCasFinder (https://crispr.i2bc.paris-saclay.fr/; Couvin et al., 2018), CRISPRminer (http://www.microbiome-bigdata.com/CRISPRminer/; Zhang et al., 2018), and CRISPRDetect (http://crispr.otago.ac.nz/CRISPRDetect/predict_crispr_array.html; Biswas et al., 2016). The following search parameters were used: minimum length of the repeated sequence, 11 base pairs (bp); maximum length of the repeated sequence, 50 bp; minimum size of spacer sequences based on repeat size, 0.6; maximum size of spacers based on repeat size, 2.5; and maximum percentage of similarity between spacers, 60% (Cruz-López et al., 2021).

The CRISPR-Cas flanking genomic regions were manually reviewed, considering the 20 000 bp sequences upstream and downstream of the arrays. The sequences, predicted structures, and functional annotations of the proteins were analyzed using the UniProt database and I-TASSER server.

#### 2.2.1 Analysis of CRISPR arrays

The matrices were extracted manually from the output file. The number, size, and location of RSs (repeated sequences) and SSs (spacer sequences) were determined. The RSs were analyzed with the CRISPRDetect program to establish the variations into RSs and associated with a consensus sequence. The SSs were analyzed using the CRISPRTarget program (http://crispr.otago.ac.nz/CRISPRTarget/crispr_analysis.html; Biswas et al., 2013) to identify the protospacer adjacent motif (PAM) and the genes associated with each spacer. The types and subtypes of the CRISPR systems were analyzed with the CRISPRmap program (http://rna.informatik.uni-freiburg.de/CRISPRmap/Input.jsp; Lange et al., 2013) using the consensus RS file as the input file.

### 2.3 Analysis of Cas proteins

The Cas protein sequences in fasta format were obtained from the RefSeq and TIGRFAM databases (ftp://ftp.ncbi.nih.gov/genomes/; http://www.jcvi.org/cgi-bin/tigrfams/index.cgi; Abby and Rocha, 2017) and were identified using the MacSyFINDER program (v 1.0.5) and makeblastdb (v2.2.28) implemented in Python (https://github.com/gem-Pasteur/macsyfinder; Abby and Rocha, 2017). According to the data obtained with this program, classification of the CRISPR–Cas systems was carried out.

### 2.4 Analysis of resistance and virulence genes

The functional annotation was updated by Prokka v1.12 (Seemann, 2014) and RAST programs (Rapid Annotation using Subsystem Technology) (Aziz et al., 2008). The antibiotic resistance genes were identified using ResFinder (https://cge.cbs.dtu.dk/services/ResFinder/; Zankari et al., 2012) and BacWGSTdb (http://bacdb.cn/BacWGSTdb/analysis_single.php; Ruan and Feng, 2016). The virulence genes were identified using the Virulence Factor Database (VFDB) (http://www.mgc.ac.cn/cgi-bin/VFs/genus.cgi?Genus=Acinetobacter; Chen et al., 2005).

The prophages were identified by PHASTER (https://phaster.ca/; Arndt et al., 2016) classified as intact (score >90), incomplete (score 70–90) or related (score <70) prophages, and with Prophage Hunter (https://pro-hunter.genomics.cn/index.php/Home/Index/index.html; Song et al., 2019) classified as active (score of 0.8), ambiguous (score of 0.5–0.8), or inactive (<0.5 score) prophages. The prophage number was determined by removing overlaps of the same prophages. The sequences recognized as related or inactive prophages were eliminated.

### 2.5 Sequence alignment and tree construction

The phylogenetic analysis was performed by VBCG (Validated Bacterial Core Genes) (https://github.com/tianrenmaogithub/vbcg, Tian and Imanian, 2023) with validated genes. The tree was inferred using an approximately-maximum-likelihood phylogenetic with FastTree.

### 2.6 GenBank accession number and data availability

The GenBank accession numbers of the genome sequences used in this study are listed in Supplementary Table 1. The data generated in this work are available at the following link: https://github.com/JetsiMancilla/System-CRISPR-Cas.

## 3 Results

### 3.1 Identification of CRISPR–Cas systems in Acb complex genomes and plasmids

The data generated with the CRISPRCasFinder, CRISPRDetect, and CRISPRRminer programs allowed the detection of CRISPR arrays (confirmed and probable) and/or Cas proteins. Only in 293 Acb complex genomes described as “complete genomes (108)” or “draft genomes (105),” which included 80 plasmids, were CRISPR–Cas, CRISPR arrays or Cas proteins identified in the following species: *A. baumannii* (150), *A. nosocomialis* (42), *A. pittii* (76), *A. calcoaceticus* (12), *A. seifertii* (11), and *A. lactucae* (2) (Supplementary Table 1).

### 3.2 Genome description and identification of ST and rSTs

The analyzed chromosomal genomes (213) had sizes between 3.4 and 4.3 Mb, and the plasmid (80) genomes had sizes between 0.004437 and 0.33 Mb (Supplementary Table 1). The genomes exhibited a GC content of 39% (±0.1). The RAST annotation showed more than 3,690 to 4,181 coding regions in the genomes, linked to between 303 and 323 subsystems. In contrast, the Prokka annotation revealed a range of 3,427 to 5,415 coding regions (https://github.com/JetsiMancilla/System-CRISPR-Cas).

The genomes included in this study were mainly from strains isolated from patients with pneumonia, nosocomial infection, wounds, urinary tract infection, bacteremia, meningitis, or septicemia (Supplementary Table 1). The strains were mainly isolated from blood (18.8%, 55/293), sputum (9.2%, 27/293), wound (4.4%, 13/293), urine (3.7%, 11/293), and the respiratory tract (3.4%, 10/293) (Supplementary Figure 1).

According to the analysis of the 293 genomes, 145 different rSTs were established with the following prevalence values: rST8482 at 8.5% (25/293), rST8237 at 3.4% (10/293), rST8274 and rST8863 at 2.4% (7/293), rST8770 at 2.0% (6/293), and rST31297 at 1.7% (5/293). The rMLST analysis confirmed that the strains belonged to the Acb complex; however, this analysis also showed that rST8378 was present in *A. baumannii* and *A. pittii* (Supplementary Table 1). In addition to the rMLST, 18 CC (Clonal Complex) with 130 STs were defined, the most frequently were: ST208 11.5% (15/130), ST231 8.5% (11/130) and ST540 6.2% (8/130), whereas 19 ST belonging to CC92 were found (Table 1, Supplementary Table 1).

**Table 1:** CRISPR-Cas systems confirmed in Acb complex genomes.

**Specie**	**ID Strain**	**rST**	**ST (Oxford)**	**CC (Clonal Complex)**	**Subtype**
*A.baumannii*	10_3	171828	1523		I-Fa
*A.baumannii*	10_4	171828	1523		I-Fa
*A.baumannii*	11W359501	8237	231	231	I-Fb
*A.baumannii*	36-1512	66014	696	696	Undefined
*A.baumannii*	3207	39401	1321		I-Fa
*A.baumannii*	7804	8777	490	110	I-Fb
*A.baumannii*	7835	131131	227		I-Fa
*A.baumannii*	9102	90872	231	231	I-Fb
*A.baumannii*	40288	8921	229	110	I-Fb
*A.baumannii*	AB43	8915	705	110	I-Fb
*A.baumannii*	AB307-0294	8237	231	231	I-Fb
*A.baumannii*	AB5075-UW	8237	945	109	I-Fb
*A.baumannii*	ab736	9295	231	231	I-Fa
*A.baumannii*	A1	8237	231	231	I-Fb
*A.baumannii*	A85	8529	781	231	I-Fb
*A.baumannii*	A388	8527	439	109	I-Fb
*A.baumannii*	A1296	68433	1469		I-Fb
*A.baumannii*	A1429	8644	1508		I-Fa
*A.baumannii*	Ab-C102	146942	1856		I-Fa, I-Fb
*A.baumannii*	AbH12O-A2	39392	924	636	I-Fa
*A.baumannii*	AC1633	163933	2089		I-Fb
*A.baumannii*	AF-401	51339	1942		I-Fa
*A.baumannii*	AR_0063	9802	205	636	I-Fa
*A.baumannii*	AR_0083	8237	231	231	I-Fb
*A.baumannii*	AR_0088	8921	229	110	I-Fb
*A.baumannii*	AR_0101	9802	124	636	I-Fa
*A.baumannii*	ATCC_19606	9295	931	110	I-Fa
*A.baumannii*	ATCC_17961	9679			I-Fa
*A.baumannii*	Ax270	8237	931	110	I-Fb
*A.baumannii*	D4	8777	218		I-Fb
*A.baumannii*	D36	9603	364	110	I-Fb
*A.baumannii*	D46	172609	498	109	I-Fb
*A.baumannii*	DA33382	8237	229	110	I-Fb
*A.baumannii*	DETAB-P2	157145	540	92	I-Fa
*A.baumannii*	DETAB-P39	9740	2209		I-Fa
*A.baumannii*	E47	126159	540	92	I-Fb, Orphan arrays
*A.baumannii*	Ex003	8237	1262		I-Fb
*A.baumannii*	FDAARGOS_533	97496	231	231	I-Fa
*A.baumannii*	FDAARGOS_1036	8954	2307		I-Fb
*A.baumannii*	HWBA8	8921	208	208	I-Fb
*A.baumannii*	MRSN 56	8237	447	92	I-Fb
*A.baumannii*	B8342	68309	2058		Orphan arrays
*A.baumannii*	OCU Ac18				I-Fa, I-Fb
*A.baumannii*	P7774	121693	2762		I-Fb
*A.baumannii*	UPAB1	90875	2055		I-Fb
*A.baumannii*	USA15	8529	490	110	I-Fb
*A.baumannii*	VB82	121693	491	109	I-Fb
*A.baumannii*	VB2486	97503	451	92	I-Fb
*A.baumannii*	WCHAB005078	79205	2162		I-Fb
*A.baumannii*	WP4-W18-ESBL-11	159126	449	109	I-Fb
*A.baumannii*	XL380	68402	1351		I-Fa
*A.baumannii*	J9	9214	718	267	Undefined
*A.baumannii*	B8300	68308			I-Fa
*A.baumannii*	FDAARGOS_540-1-plasmid	97497	540	92	Orphan arrays
*A.baumannii*	OCU_Ac18-plasmid	178505	2279		Orphan arrays
*A. baumannii*	DB053-plasmid	201153	1358		I-Fb
*A.nosocomialis*	AB6	79222	2617		I-Fa
*A.nosocomialis*	AB7	79222	2629		I-Fa
*A.nosocomialis*	AC13	169922	279	147	I-Fa
*A.nosocomialis*	AC15	169922	Undefined		I-Fa
*A.nosocomialis*	AC25	169922	Undefined		I-Fa
*A.nosocomialis*	AC1537	31297	3322		I-Fv
*A.nosocomialis*	AC1781	126169	1343		I-Fa
*A.nosocomialis*	TG19596	31328	2154		Orphan arrays
*A.nosocomialis*	TUM15142	68464	2598		Orphan arrays
*A.nosocomialis*	TUM15284	79222	2078		I-Fa
*A.nosocomialis*	TUM15298	79222	2605		I-Fa
*A.pittii*	ANC_4050	8370	1069		Undefined
*A.pittii*	MCR53	66041	1818		I-Fb
*A.calcoaceticus*	ACa13	182908	1068		I-Fa
*A.calcoaceticus*	DSM_30006	Undefined	Undefined		I-Fa
*A.calcoaceticus*	GK1	Undefined	Undefined		I-Fa, Orphan arrays
*A.calcoaceticus*	GK2	68443	Undefined		I-Fa, Orphan arrays
*A.calcoaceticus*	JUb89	Undefined	Undefined		I-Fv, Orphan arrays
*A.calcoaceticus*	KCTC_2357	68441	Undefined		I-Fa
*A.calcoaceticus*	NCTC12983	8231	1040	92	I-Fa
*A.calcoaceticus*	TG19588	8699	1042		I-Fa

### 3.3 CRISPR–Cas subtypes I-Fa, I- Fb and I-Fv were the most frequent

The CRISPR arrays characterized by RSs (repeat sequences) and SSs (spacer sequences) were confirmed in 26.30% (77/293) of the Acb complex genomes (including three plasmids). Interestingly, more than one CRISPR array was detected in the same genome or plasmid, being a total of 85 CRISPR arrays. Briefly, 70.2% (60/85) of the CRISPR arrays corresponded to *A. baumannii*, 14.3% (12/85) to *A. calcoaceticus*, 13.1% (11/85) to *A. nosocomialis*, and 2.4% (2/85) to *A. pittii* (Supplementary Table 2).

**Figure 2:**
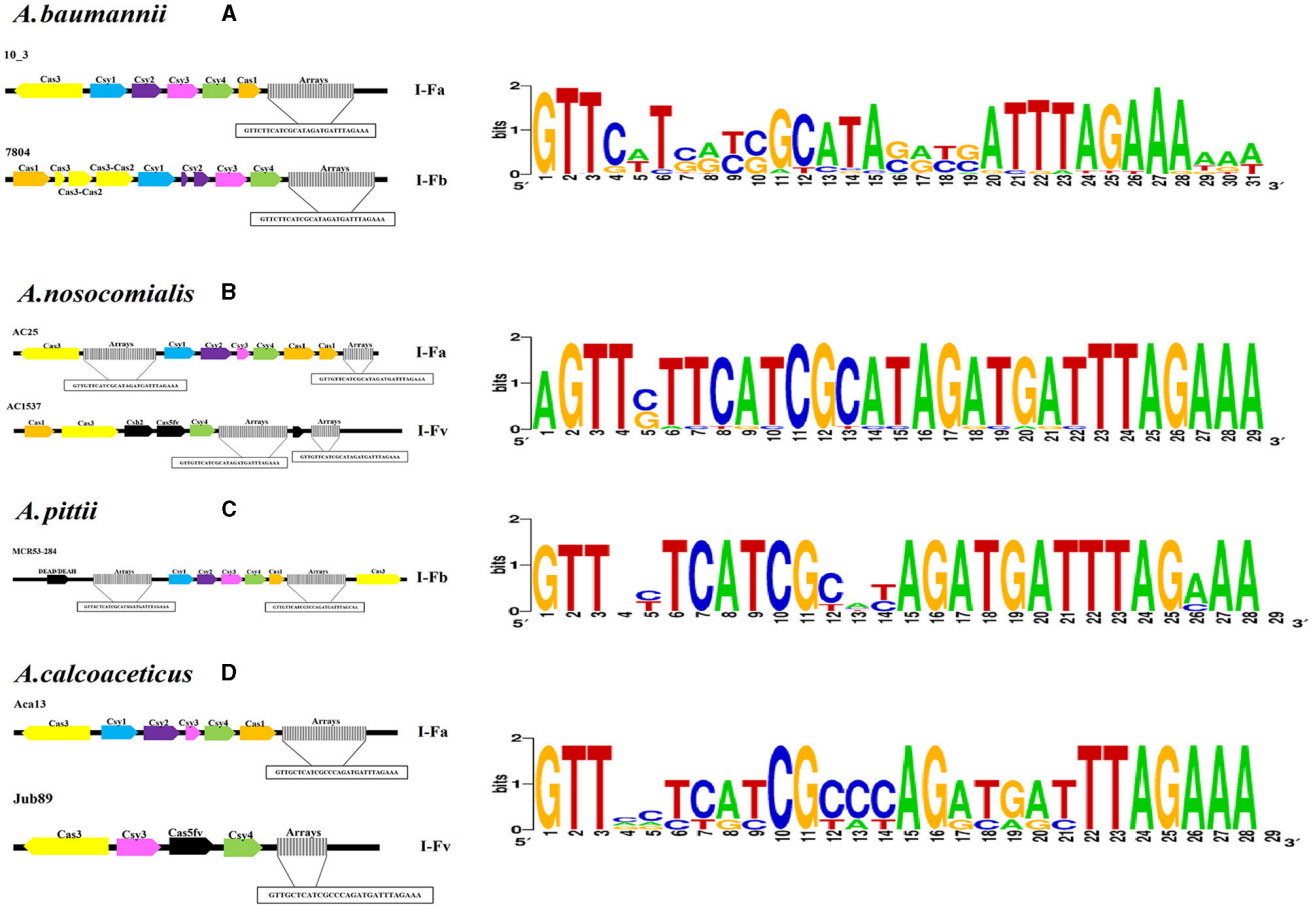
Identification of CRISPR–Cas systems through bioinformatics tools. Characteristics of the CRISPR–Cas systems detected in Acb complex chromosomes showing the SR consensus sequence and its variations. **(A)** Subtypes of CRISPR–Cas systems identified in *A. baumannii* chromosomes. **(B)** Subtypes of CRISPR–Cas systems identified in *A. nosocomialis* chromosomes. **(C)** Subtypes of CRISPR–Cas systems identified in *A. pittii* chromosomes. **(D)** Subtypes of CRISPR–Cas systems identified in *A. calcoaceticus* chromosomes. A graphic of the sequences was made with WebLogo (https://weblogo.berkeley.edu/., Crooks et al., 2004).

The number of RSs ranged from three to 158, with lengths of 24 to 29 bp in the CRISPR arrays. The RSs consensus sequences obtained by CRISPRDetect were compared with the RS sequences identified in this study (Figure 2, Supplementary Table 2).

The RS consensus sequences were clustered into 14 distinct groups, with the sequence “GTTCATGGCGGCATACGCCATTTAGAAA” being the most frequent. Interestingly, the consensus RS described in this study showed a minimum of four variations and a maximum of 11 variations compared with the consensus type I-Fb RS reported previously (Karah et al., 2015). Additionally, four RSs were detected in three plasmids, only one of which was closely related (IX) to the RS identified on chromosomes (Supplementary Figure 2).

Furthermore, 2,768 SSs with lengths between 29 and 38 bp were identified in this study. Interestingly, the *A. baumannii* and *A. calcoaceticus* genomes had the greatest numbers of SSs (Supplementary Table 3).

**Figure 3:**
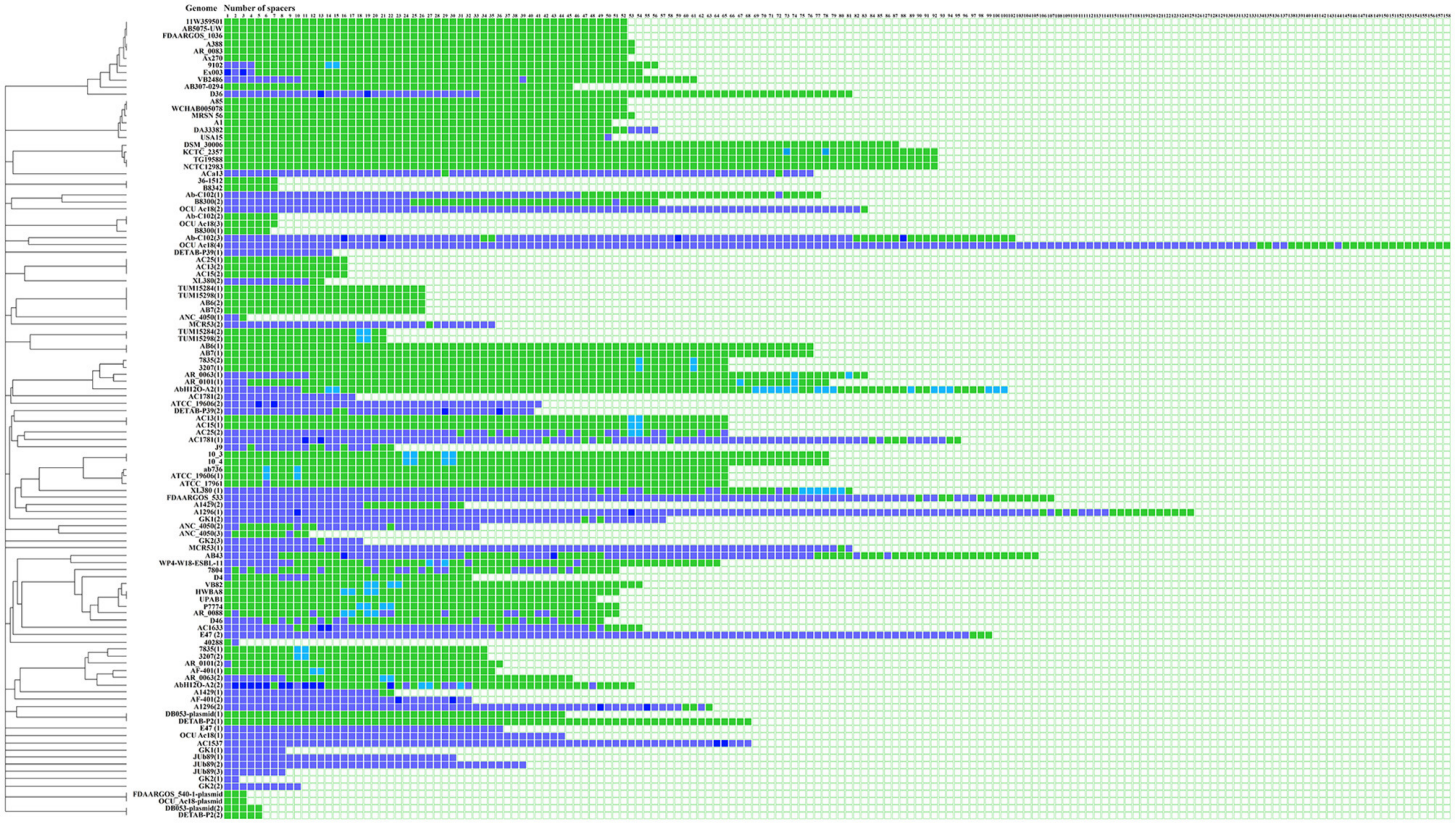
Identification of SSs in CRISPR–Cas systems. The number of spacers is indicated on each confirmed array. Shared SSs between genomes are highlighted in light green boxes, and unique spacers are shown in purple boxes. SSs that appear multiple times in the array and are shared between genomes are represented by light blue boxes. Unique SSs that appear multiple times in the array are shown in navy blue. Visualization was performed with the iTol program (Letunic and Bork, 2021).

The SSs were clustered into 30 groups according to their sequence. A total of 30.5% (845/2,768) of the SSs were shared among the genomes, and 69.4% (1,923/2,768) of the SSs were exclusive, which means that they were not commonly found in the other analyzed genomes. Interestingly, in 45.0% (36/85) of the CRISPR arrays, the same spacer was identified two or more times (Figure 3, Supplementary Table 3).

The Acb complex genomes showed the presence of CRISPR arrays, and adjacent sequences corresponding to the six *cas* genes (*cas1, csy1, csy2, csy3, csy4*, and *cas3*) identified subtypes I-Fa, I- Fb and I-Fv (Supplementary Figures 3–12). The size of the CRISPR–Cas system in the genome ranged from 4 617 to 750 328 bp (Table 1).

The CRISPR–Cas systems identified in *A. baumannii* genomes were classified into the I-Fa 33.3% (20/60) and I-Fb 55% (34/60) subtypes, which included one plasmid (Supplementary Figures 3–8, Supplementary Table 4). Additionally, 3.3% (2/60) of the CRISPR systems were considered undefined and displayed CRISPR arrays associated with the DEAD/DEAH box helicase motif (36-1,512) and the *cas3* gene (J9) (Supplementary Figure 10, Supplementary Table 4). Four solitary (orphan) arrays were defined among the *A. baumannii* genomes and plasmids (Supplementary Figure 9).

The *A. nosocomialis* CRISPR–Cas subtypes I-Fa and I-Fv were detected in 72.3% (8/11) and 9.1% (1/11) of the genomes, respectively. Two orphan CRISPR arrays were identified at loci other than the *cas2* gene and DEAD/DEAH box helicase motif in the two genomes (Supplementary Figure 10).

Two systems were confirmed to be present in the *A. pittii* genomes, and only one system was found to be complete, with two arrays associated with a set of *cas* genes characteristic of the I-Fb subtype; however, the remaining system in the *A. pittii* genomes presented three small arrays and only the *cas3* gene (Figure 2, Supplementary Figure 11).

Overall, 87.5% (7/8) and 12.3% (1/8) of the *A. calcoaceticus* genomes exhibited the CRISPR–Cas I-Fa and I-Fv subtypes, respectively (Supplementary Figure 12).

### 3.4 Probable CRISPR arrays and *cas* genes

The 200 potential CRISPR arrays were determined with the CRISPRCas Finder program. These arrays were associated with 44 consensus RSs and included between two and six RSs with lengths of 18–36 bp (Supplementary Table 4). In Acb complex genomes and plasmids, putative CRISPR arrays were found in 56.8% (121/213) and 98.8% (79/80), respectively.

The putative CRISPR array genes upstream and downstream encoded 7.5% (15/200) of the *A. baumannii* genome to the *cmr5* gene (CRISPR type III-B/BAMP) and 3% (6/200) to the DEAD/DEAH box helicase motif (Supplementary Table 4). Interestingly, the RS “GGACAAAGCGTTGCTTTGTTTATCCTC” sequence identified in two putative CRISPR arrays in *A. baumannii* genomes has been described in the CRISPR–Cas type I-B system.

Furthermore, the sequences predicted by CRISPR–Cas Finder were similar (>60%) to those of the *cas3* gene in 65 genomes, corresponding to *A. nosocomialis* (36.9%, 24/65), *A. pittii* (60%, 39/65), and *A. calcoaceticus* (3.7%, 2/65). In the *A. pittii* genome, the *csm3* and *cmr5* genes were within the loci of the CRISPR–Cas III-A and III-B systems, respectively; however, CRISPR arrays were not found in these genomes (Supplementary Table 5).

### 3.5 The flanking regions in CRISPR–Cas systems were linked to Cap4 and the toxin-antitoxin system

**Figure 4:**
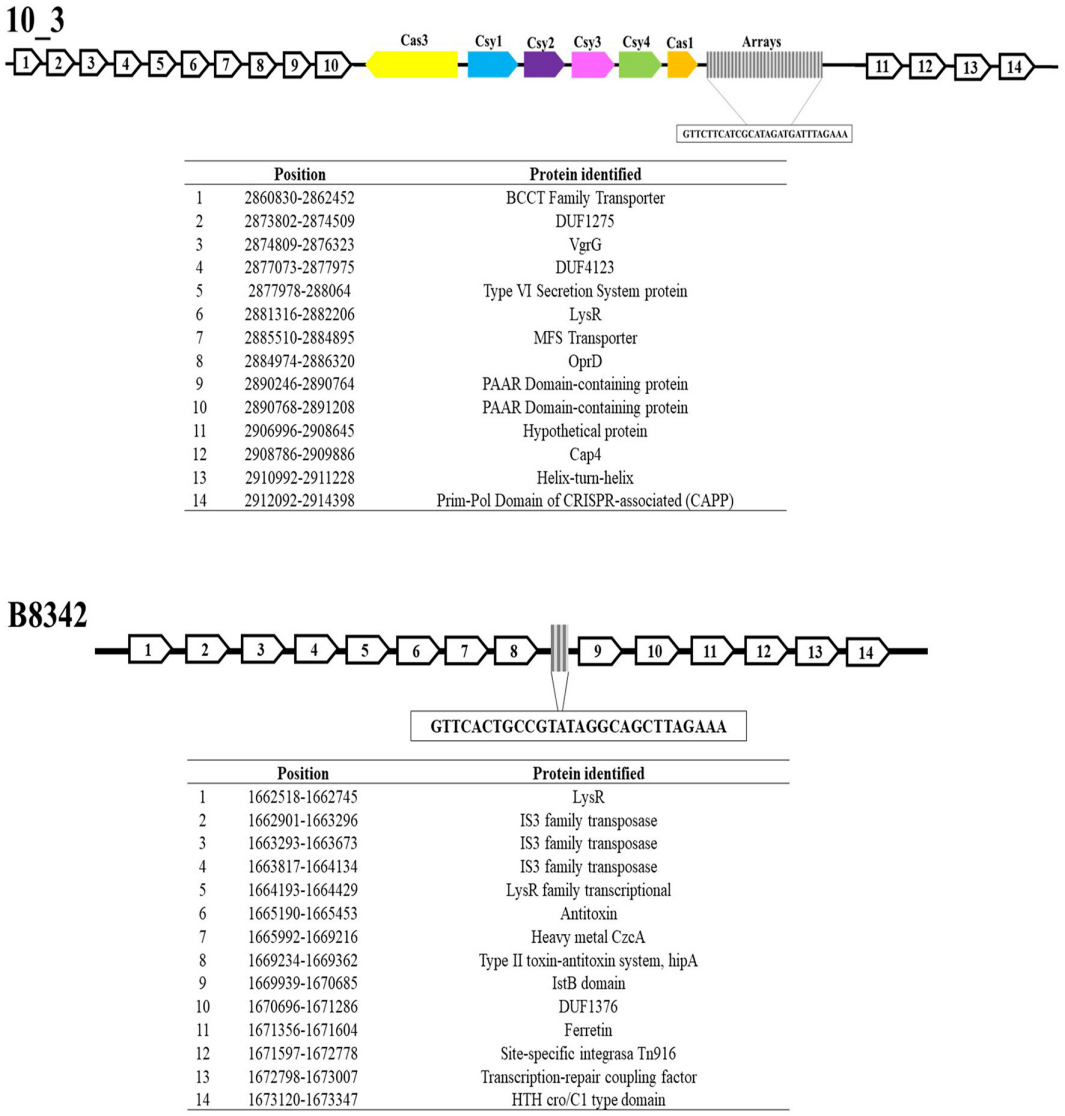
CRISPR-flanking genomic regions of the Acb complex. This figure shows the regions associated with the arrays, highlighting the regions encoding Cap4, CAPPSs, transposons, and toxin–antitoxin systems.

Analysis of genes upstream and downstream (the flanking regions) revealed sequences that encode Cap4 proteins, which can recognize cyclic oligonucleotide-based antiphage signaling systems (CBASSs), in two *A. baumannii* CRISPR arrays (the 10_3 and 10_4 genomes). Similarly, CRISPR-associated primase-polymerases (CAPPs) were identified in both genomes (Figure 4, Supplementary Figure 12).

On the other hand, 9.4% (8/85) of the CRISPR–Cas systems exhibited sequences associated with the TIGR03984 protein family, and 4.7% (4/85) exhibited sequences associated with toxin–antitoxin systems. Sixty percent (6/10) of the orphan arrays displayed sequences encoding IS3, IS5, and IS6 transposases, ammonium transporters and glutamate genes (Figure 4).

### 3.6 Prophages were identified in the Acb complex

Prophage genomes were identified in 68.94% (202/293) of the Acb complex genomes. Among these genomes, 79.8% (161/202) exhibited 1 to 8 complete prophages, and 20.2% (41/202) exhibited incomplete prophages. The prophage genomes found in this study (complete and incomplete) have been reported for several bacterial genera, such as Acinetobacter, Enterobacter, Salmonella, Aeromonas, Moraxella, Pseudomonas, Escherichia, and Ralstonia (Supplementary Table 6, Supplementary Figure 14). The 67.5% (52/77) of Acb complex genomes with confirmed CRISPR-Cas systems displayed at least one intact prophage; the 48.07% (25/52) were associated with the prophage PHAGE_Acinet_YMC11/11/R3177, and 46.15% (24/52) were linked to the prophage PHAGE_Acinet_Bphi_B1251 (Supplementary Figure 14).

**Figure 5:**
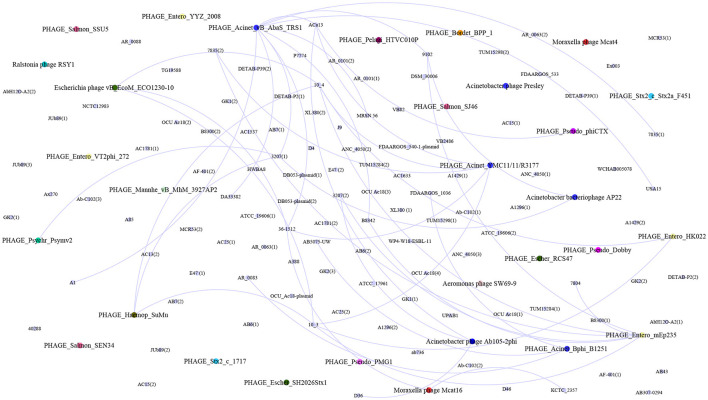
Correlation between spacers and intact prophages in the genomes. Briefly, the correlations and interactions between intact prophages and spacers targeting them are described. The purple circles represent the genomes with CRISPR–Cas systems, and the colored circles represent the intact prophages identified among the studied genomes. The networks show the interactions among the genomes that carry spacers associated with the identified prophages. The analysis and visualization of the networks were carried out with Gephi 0.10.1 (https://gephi.org/, Bastian et al., 2009).

Forty-two percent (1180/2760) of the SSs were associated with regions of prophages and plasmids belonging to the Acinetobacter genus, as well as 22 other genera, including mainly Staphylococcus, Aeromonas, Bacillus, Pseudoalteromonas, Salmonella, and Escherichia. The spacer target was a specific sequence within the prophages; however, different SSs matched the same bacteriophage (Supplementary Table 7). While some genomes possess SS that provides immunity against specific prophages, the detection of the same SS in different CRISPR arrays has been identified (Figure 5).

### 3.7 Anti-CRISPR proteins identified in bacteriophages

**Figure 6:**
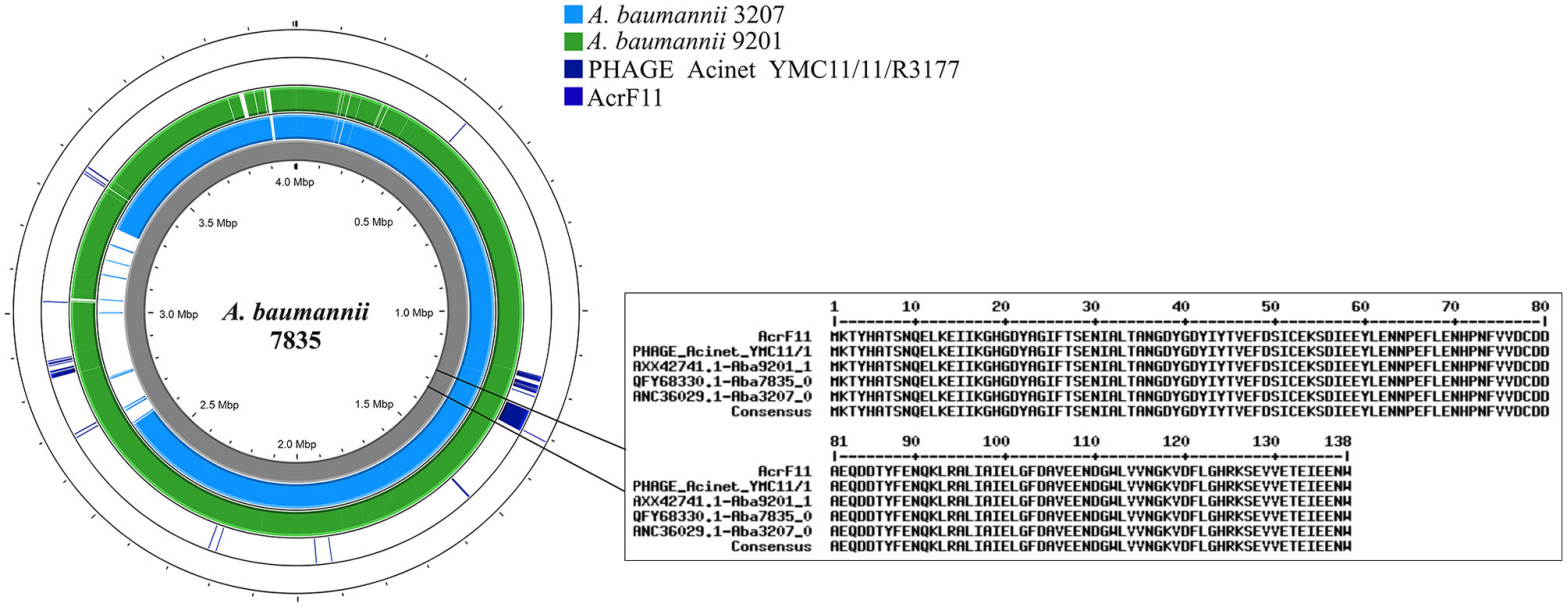
Anti-CRISPR proteins encoded in the genomes of the Acb complex. Identification of the AcrF11 protein in the LAC4, 7835, 3207, and 9201 genomes. The position of the protein coincides with the position of the PHAGE_Acinet_YMC11/11/R3177 in the genomes of the Acb complex.

PHAGE_Acinet_YMC11/11/R3177 was identified in three genomes of *A. baumannii* (7,835, 3,207, and 9,201). The AcrF11 anti-CRISPR sequence showed 100% identity with Aba7835_06455 (QFY68330.1), Aba3207_05070 (ANC36029.1), and Aba9201_17950 (AXX42741.1). In the LAC4 genome, 95% identity was confirmed for the AcrF11 sequence, but this sequence was not found in an intact phage (Figure 6, Supplementary Table 8). The search for anti-CRISPR systems led to the identification of 15 *A. pittii* genomes and sequences related to DUF2829 domains associated with AcrIIA7 (https://github.com/JetsiMancilla/System-CRISPR-Cas).

### 3.8 Toxin-antitoxin systems and antibiotic resistance elements associated with *A. baumannii* plasmids

Analysis of the Acb complex genomes revealed genes involved in colonization and virulence, including genes associated with adherence, biofilm formation, immune evasion, iron absorption, regulation, quorum sensing, and serum resistance. The genes encoded on the chromosome, including the OmpA protein involved in adhesion, invasion, persistence, and dissemination, were detected in 96.7% (206/213) of the genomes, and the genes encoding efflux systems (AdeFGH pumps), 82.6% (176/213) of the Csu fimbriae and 97.2% (207/213) of the PNAG polysaccharides were associated with the formation of biofilms.

Only 68.1% (145/213) of the genes encoded the Bap protein, which also participates in biofilm formation. On the other hand, 97.9% (208/213) of the Acb complex genomes encoded genes related to two-component systems and quorum sensing proteins involved in biofilm formation and cell adhesion (BfmRS). In the Acb complex genome, operons related to capsule Lpx and LPS were identified in 84.0% (179/213). Interestingly, 72.8% (155/213) of the genomes of *A. baumannii, A. pittii*, and *A. nosocomialis* contained genes associated with iron acquisition and acinetobactin biosynthesis (Supplementary Table 9).

Toxin-antitoxin systems were identified in only 12.5% (10/80) of the plasmids. Twenty percent (16/80) of the genes were encoded to the stress response proteins, and 8.7% (7/80) were associated with resistance to toxic compounds.

Among the chromosomally encoded resistance genes, 28.6% (61/213) were related with aminoglycoside resistance, 69.0% (147/213) with beta-lactam resistance, and only 20.2% (43/213) with genes linked to tetracycline resistance.

The Acb complex plasmid analysis revealed genes associated with resistance to aminoglycosides, antagonists of the folate pathway, beta-lactams, tetracyclines, phenicols, macrolides, and polymyxins. Briefly, 12.5% (10/80) of the genes were related to macrolide resistance [*msr(E)* and *mph(E)*] and encoded efflux pumps. Moreover, 3.8% (3/80) of the genes conferred resistance to streptomycin (*str*A and *str*B), gentamicin [ant (3”) Ia], and tobramycin. Regarding the genes encoding resistance to beta-lactams, 18.8% (15/80) of the plasmids contained *bla*_OXA − 23_, *bla*_OXA − 24_, *bla*_OXA − 58_, *bla*_OXA − 72_, and *bla*_IMP − 1_ carbapenem resistance genes, such as those conferring resistance to imipenem, meropenem and doripenem.

The cephalosporin resistance genes *bla*_ADC − 25_, *bla*_PER − 1_, and *bla*_NDM − 1_ were identified in one plasmid of *A. baumannii* and *A. pittii* (Supplementary Table 9).

Several regions encoding copper ATPases, copper chaperones, regulatory proteins, and copper oxidases identified in plasmids have also been associated with the transport and oxidation of copper. The data suggested that these elements interfere with the processes of colonization and immune evasion in *A. baumannii*; however, these elements were located only in plasmids from *A. seiferttii* and *A. pittii*. Our data indicate that *A. seiferttii* and *A. pittii* plasmids encode more resistance mechanisms than *A. baumannii* plasmids.

## 4 Discussion

*A. baumannii* has been implicated in healthcare-associated infections (HCAIs), such as ventilator-associated pneumonia, meningitis, bloodstream infections, and urinary tract infections. *A. baumannii* together with *A. pitti, A. nosocomialis, A. seiferttii*, and *A. lactucae* belongs to the Acb complex. *A. pitti, A. nosocomialis, A. seiferttii*, and *A. lactucae* also cause infections and have been associated with resistance to multiple drugs (Fitzpatrick et al., 2015; Li et al., 2021; Alonso et al., 2023; Bajaj et al., 2023; Kang et al., 2023). *A. calcoaceticus* is mainly found in environmental samples and may also carry antibiotic resistance determinants (Al Atrouni et al., 2016).

The use of bioinformatics tools and the availability of genome sequences have facilitated the analysis and characterization of *A. baumannii* genomes. These studies have contributed to understanding *A. baumannii* genome dynamics and the functions of its different genetic determinants, including its response to selective environmental pressure during the evolutionary process. The analysis of *A. baumannii* genomes has allowed us to explore the presence of repeated sequences associated with CRISPR–Cas systems. However, these systems have yet to be investigated in other members of the Acb complex. The matrices that make up the repeated sequences associated with CRISPR Cas systems belong to types I-F, characterized by the presence of the *cas1, cas2, cas3, cas5, cas6*, and *cas7* genes (Karah et al., 2015; Mangas et al., 2019; Yadav and Singh, 2022).

The CRISPR–Cas system was found in one plasmid because it has been established that CRISPR–Cas systems in plasmids provide bacteria with similar efficacy in protecting against bacteriophage infections compared to those encoded in the chromosome (Siedentop et al., 2024). Additionally, we identified other plasmids characterized by a series of orphan arrays. This phenomenon has been observed in plasmids from other bacteria and archaea, where systems on plasmids are often incomplete or composed of small orphan arrays (Pinilla-Redondo et al., 2022). The prevalence of CRISPR–Cas systems in these genomes may also be associated with the evolution and adaptation of these species in diverse environments (Westra et al., 2019).

The availability of several bioinformatic tools (online and for command-line use) has facilitated the identification of CRISPR–Cas systems. This study identified and confirmed 85 CRISPR arrays and *Cas* genes according to the CRISPRCasFinder, CRISPRDetect, and CRISPRminer programs.

In this study identified CRISPR arrays or *cas* gene harbored subtypes I-B and III-B, as previously described by other authors (Yadav and Singh, 2022). Also, CRISPR arrays or genes associated with subtypes III-A and III-B in *A. pittii* were described for the first time. These systems are considered non-functional. However, there are reports suggesting that these small arrays could be evolutionarily preserved (Shmakov et al., 2023). Various phenomena could explain the isolation of these arrays; one possibility is the emergence of *de novo* arrays, or it could be that their acquisition is linked to transfer through mobile elements (Westra et al., 2019; Shmakov et al., 2023), which could explain the presence of transposases adjacent to these arrays in our study.

An interesting aspect with CRISPR-arrays was their diversity. The RSs exhibited a clear modularity; even if it's not preserved in all the identified systems. Although these sequences are not directly associated with the exogenous material that makes up the system, it is important to demonstrate their modularity, since they play a crucial role in the marking and organization of the spacer sequences, as well as in the expression and interference processes (Nethery et al., 2021).

RSs also plays a crucial role in marking and organizing spacer sequences in genomes (Nethery et al., 2021). RSs sequences can exhibit modularity; that is, they are uniform and repeated throughout the CRISPR array (Yair and Gophna, 2019). The diversity of the RSs may also be related to the diversity of spacer sequences identified.

Spacer sequences in CRISPR–Cas systems provide information on the prior exposure of bacteria to various elements (Garrett, 2021). Our data showed that the analysis of these sequences corresponded to spacers and regions of interest, thereby ensuring potential immunity against prophages and plasmids, as described by other authors (Maniv et al., 2016).

Analysis of SS in the CRISPR-Cas system revealed a correlation between the identified bacteriophages in the genomes and those for which the bacteria store information within the spacers of the CRISPR-Cas system. Furthermore, the existence of genome groups is observed, which, although not sharing identical spacer sequences, may contain information related to the same prophage.

The variability of intact prophages in Acb complex genomes analyzed in this study suggest diversity. When the environmental bacteriophage diversity is high, CRISPR–Cas systems confer an advantage to bacteria harboring these gene elements. As already described, spacers can be effective against a specific bacteriophage fraction. In this context, the low diversity of prophages among genomes suggests that those carrying CRISPR–Cas systems confer greater fitness than those lacking them (Zaayman and Wheatley, 2022).

The identification of CRISPR–Cas systems involves exploring the flanking regions of the genome. Importantly, these flanking regions in all genomes carried by CRISPR–Cas systems were not always conserved. Our study revealed that the flanking regions encoded to Cap4 proteins associated with clustered bacterial immune systems (CBASSs) (Lowey et al., 2020), which interfere with the replication of prophages, providing bacteria with a protective mechanism against bacteriophage infections. Our data suggested that there could be genomes that harbor more than two defense mechanisms against bacteriophages in parallel, leading to a decrease in the fitness of the bacteria. In contrast, other authors have proposed that a CRISPR–Cas system in association with another mechanism enhances the fitness of bacteria; therefore, two systems can coexist, i.e., CRISPR–Cas systems, restriction-modification (RM) systems, and Argonaute systems (Makarova et al., 2009; Oliveira et al., 2014; Lisitskaya et al., 2018).

Interestingly, bacteria carrying CRISPR–Cas systems have the ability to defend themselves against infection by bacteriophages. However, the bacteriophages have mechanisms that allow them to evade the action of CRISPR–Cas systems. Recent data indicate that these elements act at the DNA level or interfere with the binding of DNA to Cas proteins (Parsaeimehr et al., 2022) and in the presence of anti-CRISPR proteins in the genomes of *A. baumannii* (Yadav and Singh, 2022). However, more specific data detailing the characterization of *A. baumannii* and *A. pitti*, where anti-CRISPR Cas proteins have also been found, are still needed. These anti-CRISPR proteins seem to have different modes of action; in *A. baumannii*, they have been shown to act via recognition of the PAM sequence, and in *A. pitti*, they act by inhibiting the activity of the Cas protein (Niu et al., 2020; Forsberg, 2023). Interestingly, this study determined how bacteriophages carry anti-CRISPR–Cas information; in contrast, other bacteria do not contain anti-CRISPR–Cas information to act against the CRISPR–Cas system.

## 5 Conclusion

This study elucidates the diversity and complexity of CRISPR–Cas systems and other defense mechanisms in strains of the Acb complex. These systems play a crucial role in the adaptation and fitness of these microorganisms in various environments. The confirmed arrays displayed size and sequence variations, with consensus sequences proving difficult to link to specific Acb complex species.

## Data availability statement

The datasets presented in this study can be found in online repositories. The names of the repository/repositories and accession number(s) can be found in the article/Supplementary material.

## Author contributions

JM-R: Conceptualization, Data curation, Formal analysis, Investigation, Methodology, Validation, Visualization, Writing – original draft. VF: Supervision, Validation, Writing – review & editing. MAC: Supervision, Validation, Writing – review & editing. SAO: Investigation, Resources, Writing – review & editing. JP-F: Investigation, Resources, Writing – review & editing. JA-G: Investigation, Resources, Writing – review & editing. JX-C: Funding acquisition, Project administration, Resources, Supervision, Validation, Writing – review & editing. AC-C: Conceptualization, Formal analysis, Funding acquisition, Investigation, Project administration, Resources, Supervision, Validation, Visualization, Writing – review & editing.
