# Supplementary material for: A bioinformatic approach to identify confirmed and probable CRISPR–Cas systems in the Acinetobacter calcoaceticus–Acinetobacter baumannii complex genomes
Source: Front Microbiol. 2024 Apr 9;15:1335997. doi: 10.3389/fmicb.2024.1335997 (PMC11035748; doi:10.3389/fmicb.2024.1335997)
Supplement: Supplementary file 10 [file Data_Sheet_1.pdf]

Supplementary Material

A bioinformatic approach to identify confirmed and probable CRISPR–Cas systems in the *Acinetobacter calcoaceticus–Acinetobacter baumannii* complex genomes

Jetsi Mancilla-Rojano[1,2], Víctor Flores[3], Miguel A. Cevallos[4], Sara A. Ochoa[2], Julio Parra-Flores[5], José Arellano-Galindo[6], Juan Xicohtencatl-Cortes[2*] and, Ariadnna Cruz-Córdova[1,2*]

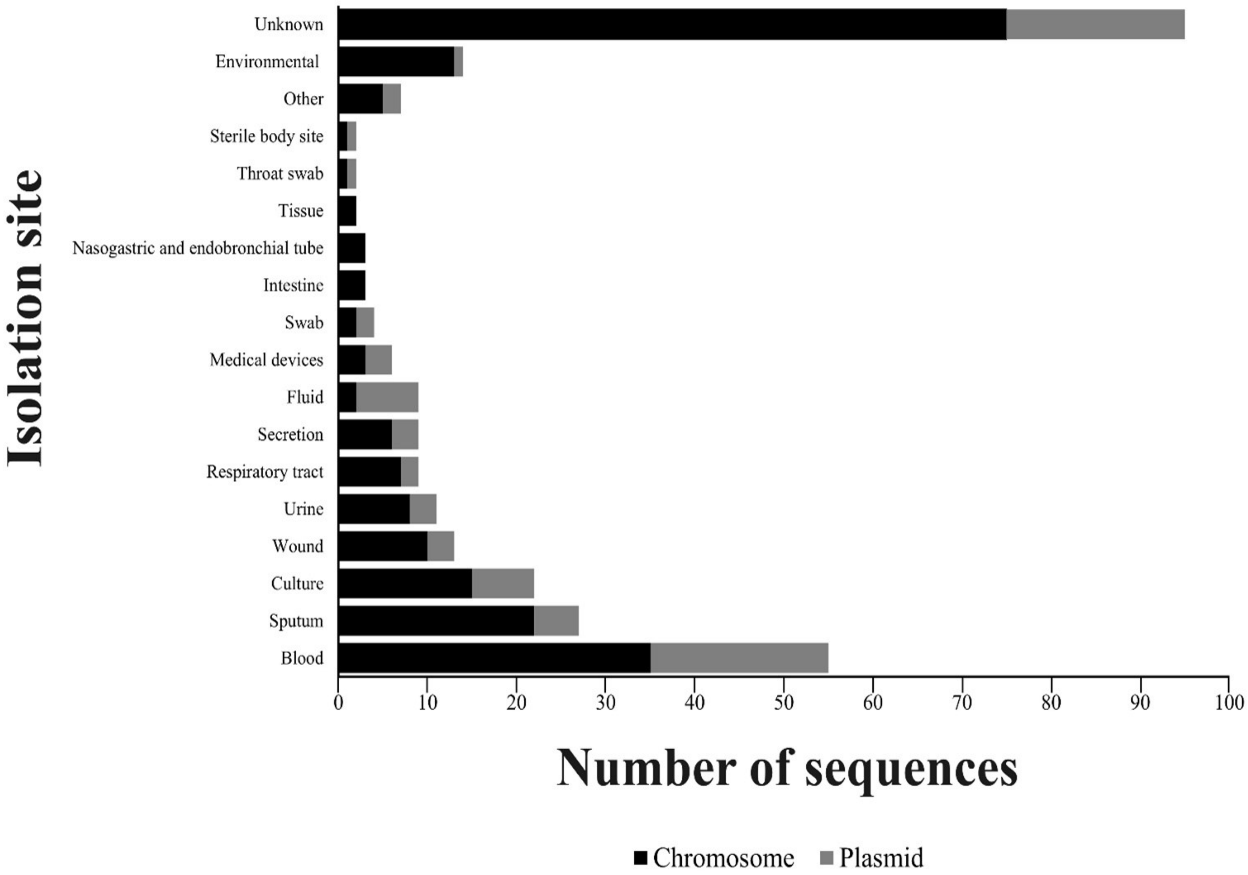

Supplementary Figure 1. Number of Sequences Analyzed of Strains Belonging to the Acb Complex. The sequences corresponding to chromosomes and plasmids of the Acb complex strains were from blood, sputum, culture (bacterial, pure, and cell culture), wound, urine, respiratory tract, secretion, fluid (bodily, ascitic, peritoneal, cerebrospinal, and pleural), medical devices (hospital and clinical sample, and stethoscope), swab (rectal swab, wound swab, and endotracheal swab), intestine (small intestine and gut), nasogastric and endobronchial tube, tissue, throat swab, sterile body site, other (pulmonary, oral cavity , tibia, hip, skin, kidney, and metagenome) and environmental (soil, marine sediment, water, rhizosphere, and terrestrial biome) samples.

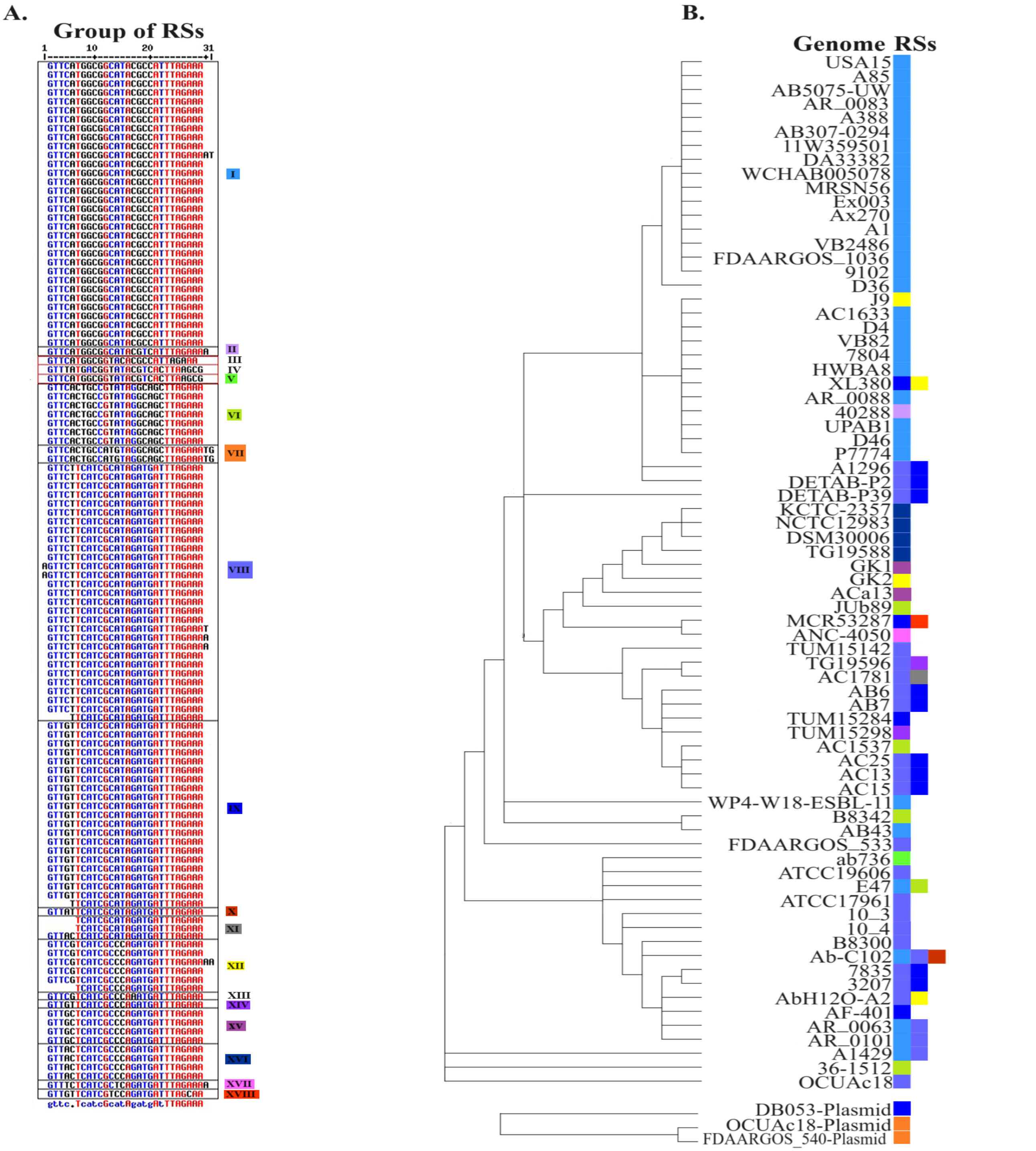

Supplementary Figure 2. A. Alignment of consensus repeat sequences. The Repeat Sequences (RSs) of the arrays confirmed in genomes of the Acb complex were aligned with the RSs reported by Karah *et al*., (2015): AYE (1), AYE (2) and ab299505 (shown in uncolored groups). The groups of RSs that were generated were represented with different colors. **B. RSs in genomes of Acb complex**. RSs associated with confirmed CRISPR-Cas systems confirmed in genomes, including three plasmids were shown. The tree was inferred using an approximately-maximum-likelihood phylogenetic with FastTree.

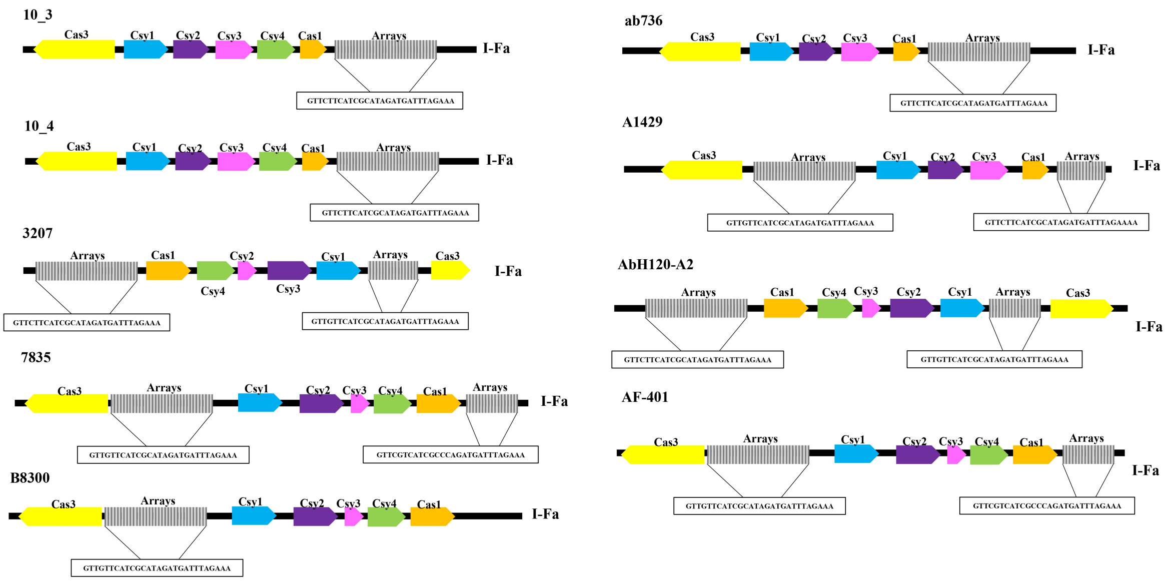

Supplementary Figure 3. Confirmed CRISPR-Cas systems. Characteristics of the subtype I-Fa detected in ***A. baumannii*** genomes 10_3, 10_4, 3207, 7835, B8300, ab736, A1429, AbH120-A2 and AF-401, showing the arrays and *cas* genes associated**.**

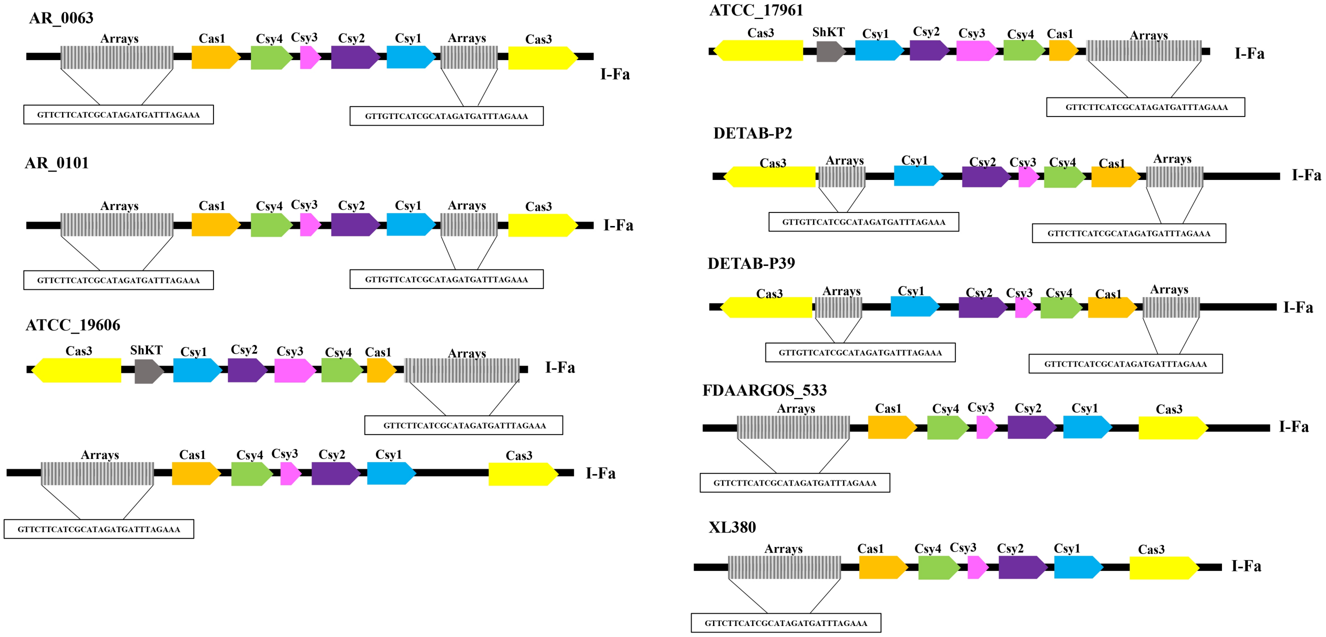

Supplementary Figure 4. Confirmed CRISPR-Cas systems. Characteristics of the subtype I-Fa detected in ***A. baumannii*** genomes AR_0063, AR_0101, ATCC_19606, ATCC_17961, DETAB-P2, DETAB-P39, FDAARGOS_533 and XL380, showing the arrays and *cas* genes associated**.**

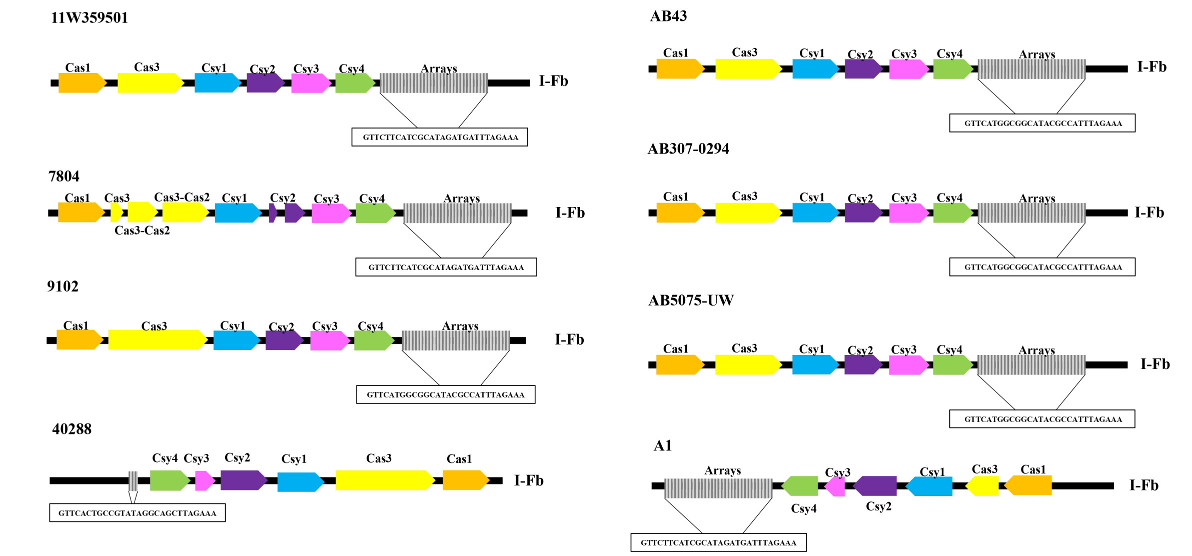

Supplementary Figure 5. Confirmed CRISPR-Cas systems. Characteristics of the subtype I-Fb detected in ***A. baumannii*** genomes 11W359501, 7804, 9102, 40288, AB43, AB307-0294, AB5075-UW and A1, showing the arrays and *cas* genes associated**.**

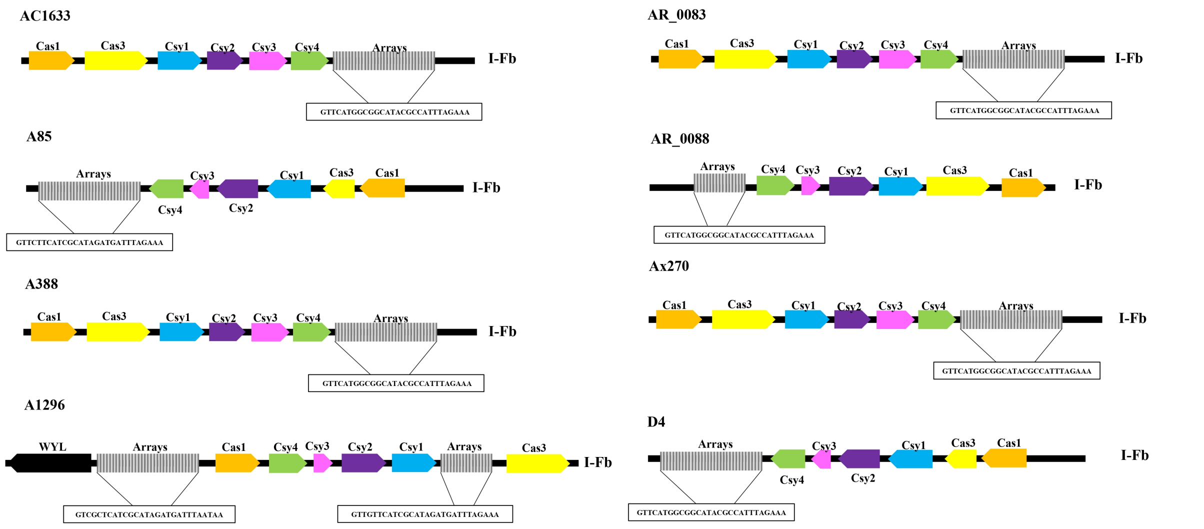

Supplementary Figure 6. Confirmed CRISPR-Cas systems. Characteristics of the subtype I-Fb detected in ***A. baumannii*** genomes AC1633, A85, A388, A1296, AR_0083, AR_0088, Ax270 and D4, showing the arrays and *cas* genes associated**.**

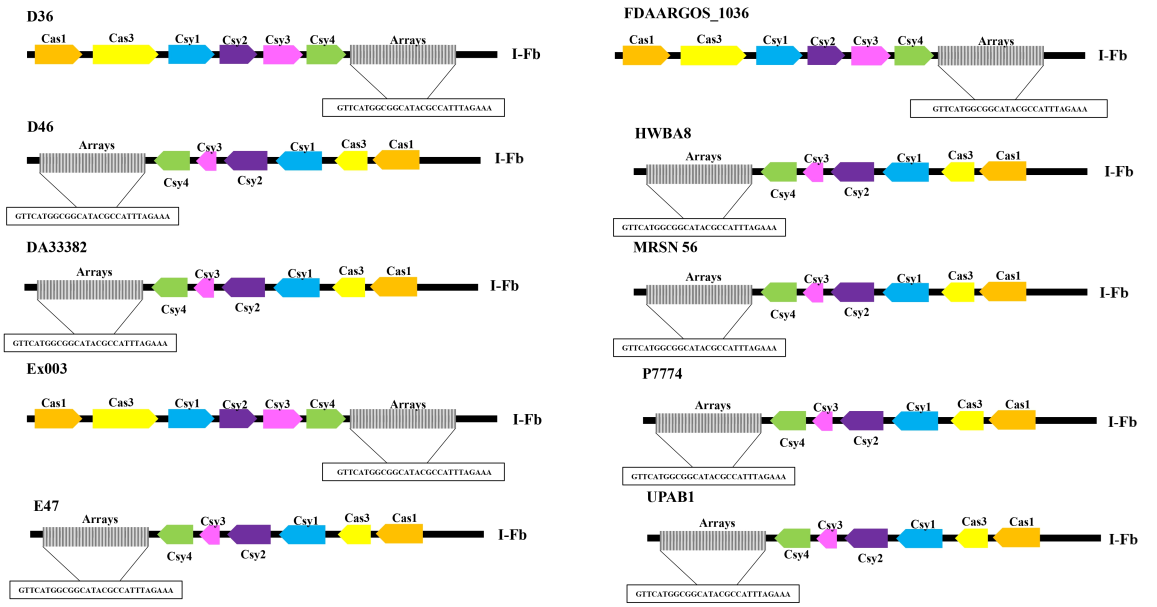

Supplementary Figure 7. Confirmed CRISPR-Cas systems. Characteristics of the subtype I-Fb detected in ***A. baumannii*** genomes D36, D46, DA33382, Ex003, E47, FDAARGOS_1036, HWBA8, MRSN56, P7774 and UPAB1, showing the arrays and *cas* genes associated**.**

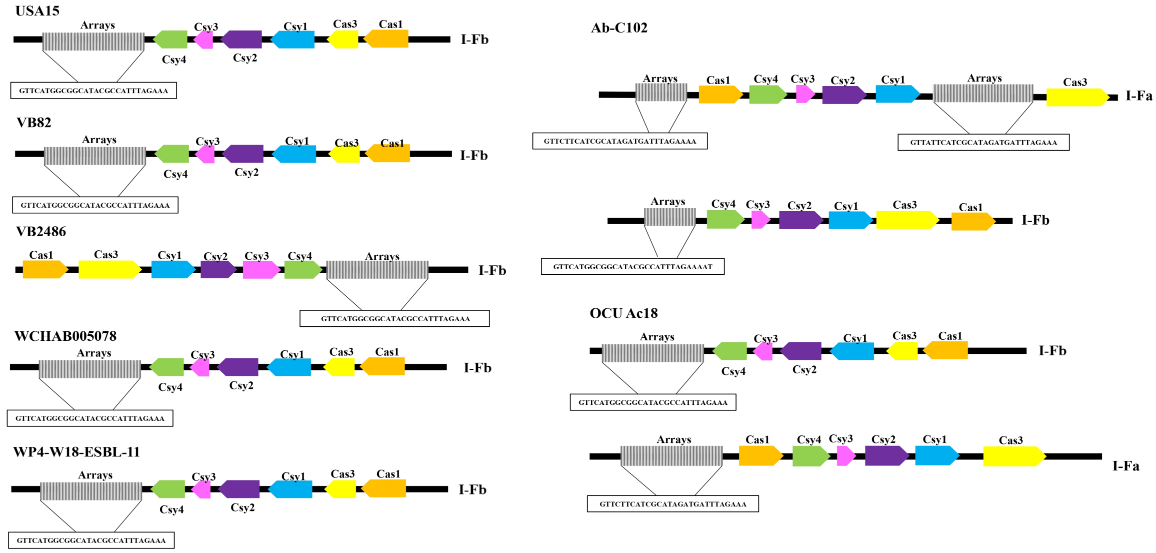

Supplementary Figure 8. Confirmed CRISPR-Cas systems. Characteristics of the subtype I-Fb detected in ***A. baumannii*** genomes USA15, VB82, VB2486, WCHAB005078 and WP4-W18-ESBL-11 Characteristics of the I-Fa and I-Fb systems, both identified in the Ab-C102 and OCU Ac18 genomes, showing the arrays and associated *cas* genes.

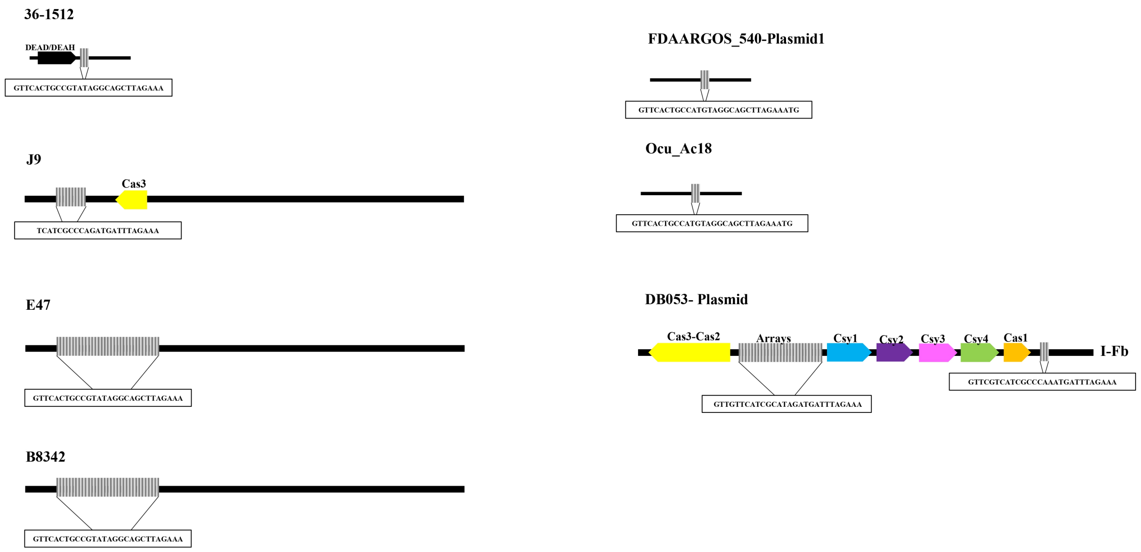

Supplementary Figure 9. Confirmed CRISPR-Cas systems. Characteristics of the systems incomplete detected in ***A. baumannii*** genomes 36-1512, J9, E47, B8342, and detected in plasmids FDAARGOS_540-Plasmid1, Ocu_Ac18, DB053-Plasmid, showing the arrays and *cas* genes associated**.**

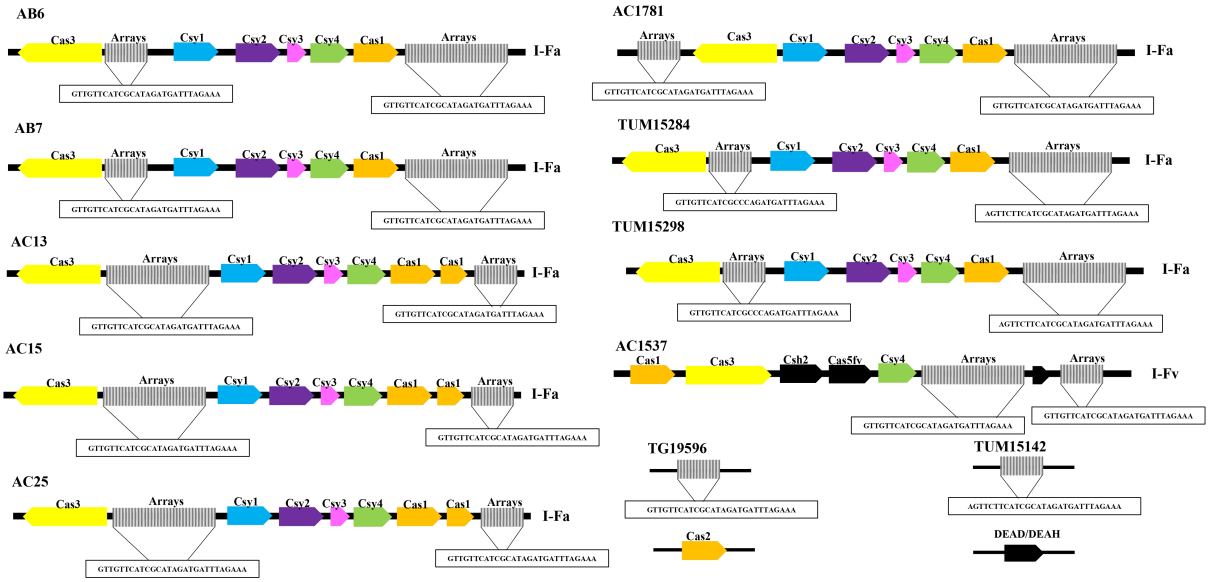

Supplementary Figure 10. Confirmed CRISPR-Cas systems. Characteristics of the subtype I-Fa and I-Fv detected in ***A. nosocomialis*** genomes AB6, AB7, AC13, AC15, AC25, AC1781, TUM15284, TUM15298, AC1537 and systems incomplete detected in TG19596 and TUM15142 genomes, showing the arrays and *cas* genes associated**.**

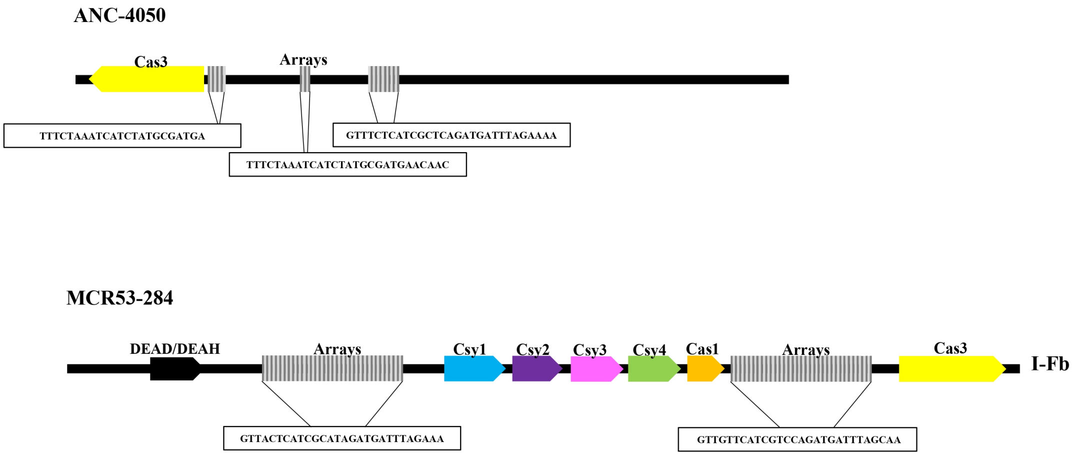

Supplementary Figure 11. Confirmed CRISPR-Cas systems. Characteristics of the systems detected in ***A. pittii*** genomes ANC-4050 and MCR53-284, showing the arrays and *cas* genes associated**.**

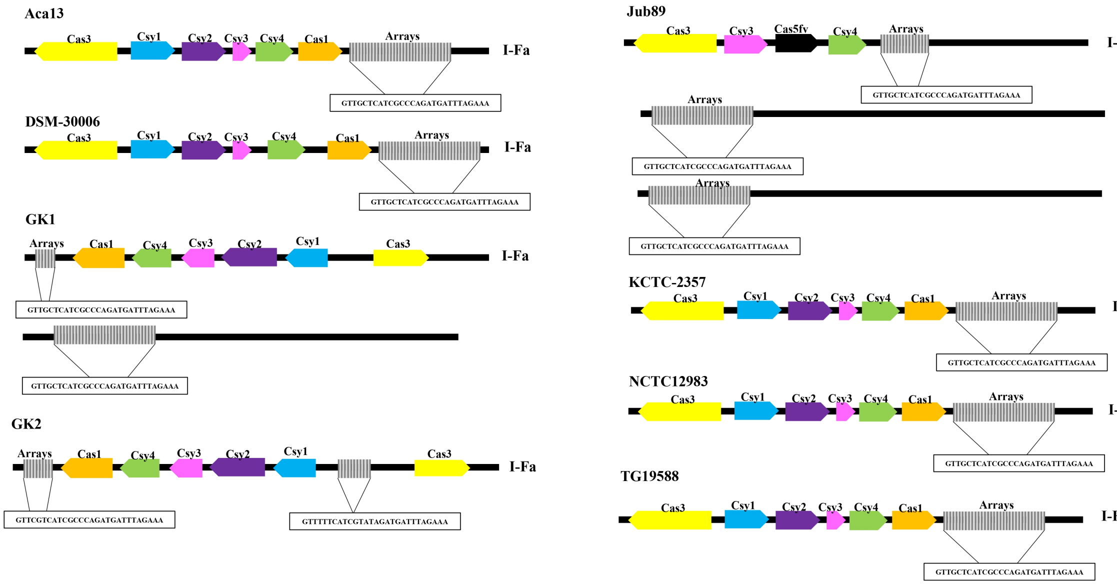

Supplementary Figure 12. Confirmed CRISPR-Cas systems. Characteristics of the subtype I-Fa and I-Fv detected in ***A. calcoaceticus*** genomes Aca13, DSM-30006, GK1, GK2, Jub89, KCTC-2357, NCTC12983 and TG19588, showing the arrays and *cas* genes associated**.**

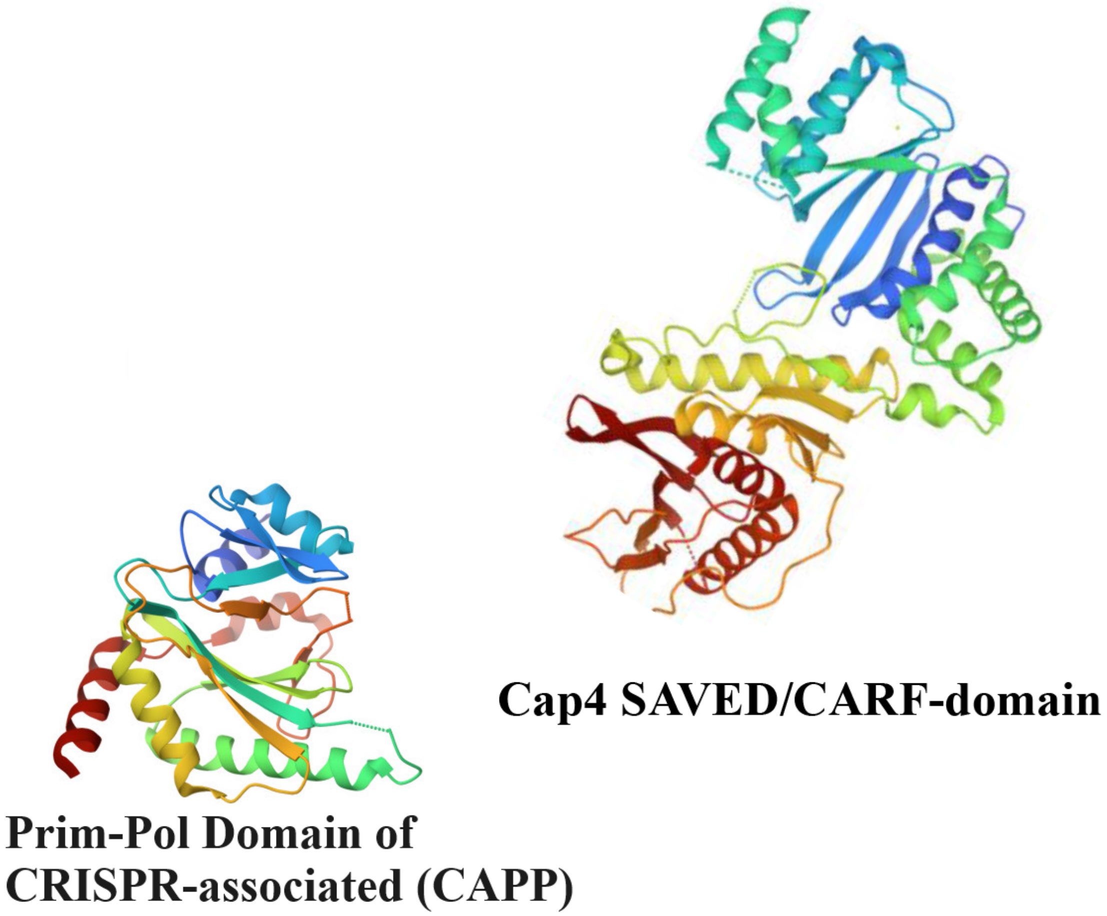

Supplementary Figure 13. Flanking proteins of the CRISPR-Cas system of the Acb complex genomes. Prediction of the three-dimensional structure of proteins surrounding CRISPR-Cas systems: Prim-Pol Domain of CRISPR-associated (CAPP) and Cap4 SAVED/CARF domain.

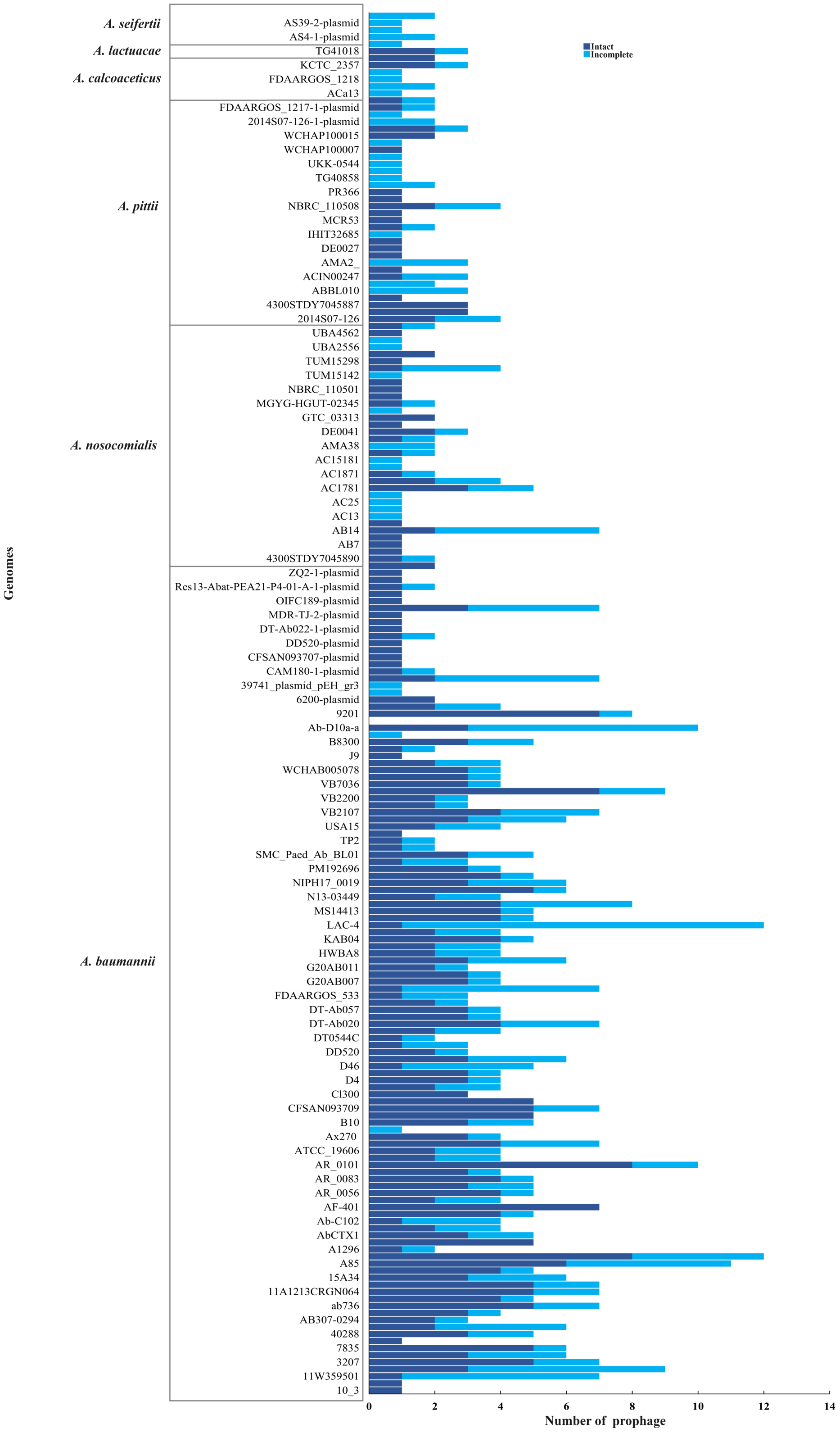

Supplementary Figure 14. Prophages in genomes of the Acb complex. The number of intact and incomplete prophages identified with the Phaster and Prophage Hunter programs in the Acb complex genomes is shown. Prophages were identified in genomes and plasmids.
